# Chronic disease and social isolation among Canadians: evidence from the 2022 Mental Health and Access to Care Survey

**DOI:** 10.24095/hpcdp.46.6.03

**Published:** 2026-06

**Authors:** Dorian DiTommaso, Fabio Robibaro, Nicholas D. Spence

**Affiliations:** 1 University of Toronto, Department of Sociology, Toronto, Ontario, Canada; 2 University of Toronto, Department of Health and Society, Toronto, Ontario, Canada; 3 University of Toronto, Department of Physical and Environmental Sciences. Toronto, Ontario, Canada; * Co-first authors.

**Keywords:** chronic disease, chronic pain, people with disabilities, social isolation, health policy

## Abstract

**Introduction:**

Chronic conditions are highly prevalent in Canada and are commonly examined as a single, aggregated exposure in population research on social isolation. Such approaches emphasize overall disease burden but make it difficult to distinguish the independent contributions of diagnostic category, chronic pain and disability. In this study, we examine these dimensions separately to assess how each is associated with social isolation among Canadian adults.

**Methods:**

Using the 2022 Mental Health and Access to Care Survey (n = 9861), the association between chronic conditions, chronic pain, and disability in relation to social support was assessed, using the Social Provisions Scale (SPS-10), applying multivariable linear regression.

**Results:**

More severe disability was negatively associated with social support (B = −0.09, 95% CI = −0.11, −0.08). Those with more functional impairments experienced lower social support which typically indicates greater social isolation.

**Conclusion:**

When examined jointly, functional disability, but not chronic disease category or chronic pain, was independently associated with lower social support. These findings indicate that social isolation among Canadian adults is more closely related to functional limitation than to diagnostic labels, underscoring the importance of function-focused approaches in research and intervention.

## Highlights

● This study examines the simultaneous impact of chronic conditions, chronic pain, and disability on social isolation using nationally representative Canadian data.

● Diagnostic labels alone are not associated with social isolation when functional limitation and pain are accounted for.

● The functional limitation is a key predictor of reduced social support, increasing social isolation.

● It is important to analyze chronic disease, chronic pain and disability together in population-level research on social isolation.

## Introduction

Chronic disease is highly prevalent among Canadian adults, carrying profound social implications like social isolation—a state of having limited social contacts or interactions.^1^^,^^2^ Social isolation is a significant public health issue, particularly in aging societies.^2^^,^^3^ In Canada, about 16% of seniors experience social isolation, with about 30% at risk due to deteriorating health.^3^ Nearly half of Canadians lived with at least one major chronic condition in 2021, and 1 in 12 had three or more; a substantial portion of the population occupies the social/health contexts in which social isolation is likely to be reported.^4^ Research on social isolation and health spans the adult life course, often emphasizing later life stages where social isolation is more prevalent, with well-documented associations between isolation, chronic conditions, and adverse health outcomes.^5^^-^^8^ Chronic conditions may not operate uniformly in relation to social isolation, highlighting the importance of examining heterogeneity across conditions when considering population-level intervention strategies.

Adults living with chronic illness are more likely to experience social isolation.^9^^,^^10^ One scoping review on the topic found that multimorbidity (e.g. having multiple overlapping chronic conditions) is linked to greater loneliness, with less evidence connecting social isolation.^10^ A later review replicated this finding among older adults.^11^ One assessment of the Canadian Longitudinal Study on Aging (n = 29 847) found that multimorbidity was linked to higher social isolation, though effects were modest.^9^ The same study shows that chronic illness modifies the impact of social roles (e.g. family, work, community engagements), on isolation with the presence of chronic disease mitigating access to these roles and their beneficial effects.^9^ Galvanizing these findings, policy reports list poor health and multiple chronic diseases as contributors to social isolation in later life.^3^^,^^12^ These works highlight the need for more population-level examinations of chronic disease and social isolation, particularly in Canada.

Research has explored chronic conditions as a homogenized category capturing the combined influence of **any** condition, despite evidence suggesting that social isolation varies substantially across conditions.^13^ Grouping diverse conditions risks obscuring important mechanisms and points of intervention. For example, heart disease and related conditions have well-documented links with social isolation that warrant further exploration.^14^ Social isolation is associated with worse cardiovascular prognoses. One meta-analysis showed that patients with poor social relationships characterized by social isolation and loneliness faced a 29% increased risk of coronary heart disease (CHD) and 32% increased risk of stroke.^15^ One cross-sectional study of 2521 Danish cardiac patients showed that loneliness and social isolation frequently co-occurred with low health literacy and high treatment burden, suggesting isolation may result from, and contribute to, difficulties in managing cardiovascular health.^16^ Similar findings exist for other conditions. Osteo- and rheumatoid arthritis are associated with increased risk of social isolation.^17^^,^^18^ One longitudinal European study found that more severe osteoarthritis was associated with a 47% increased risk of becoming socially isolated, even after adjusting for sociodemographic factors as well as depression and mobility.^19^

Chronic pain and functional disability may link chronic conditions to social isolation. Pain can be a direct symptom (e.g. arthritis) or the result of complications of illness (e.g. diabetic neuropathy or cancer). Individuals with chronic pain are vulnerable to social isolation, which may exacerbate a decline in mobility.^20^ Pain is often tied to social isolation through multiple chronic conditions with emphasis on musculoskeletal pain.^21^^-^^23^ To understand how chronic conditions uniquely affect social isolation, disaggregated individual chronic conditions (e.g. CVD, arthritis) should be assessed simultaneously alongside comorbidities like pain and functional disability.

Not all mechanisms of chronic conditions are physical. For example, cancer diagnosis and treatment disrupts daily routines and alters social identity. A 2023 American Cancer Society survey found that over half of cancer survivors felt more socially isolated, citing fatigue, pain, and fear of burdening others.^24^ While some reported closer family ties, cancer was generally linked to heightened loneliness and isolation with implications for quality of life and survival.^24^ Changed social identity and its interconnectivity with isolation in relation to chronic conditions is not unique to cancer. Chronic mental health conditions also exhibit an association with social isolation, with 53 studies in an umbrella review finding consistent associations between loneliness, isolation, and diagnoses such as depression and psychosis, though the individual review quality was low.^25^^,^^26^ Stigma compounds this phenomenon, with one qualitative study of young adults with depression describing stigma as a driver of secrecy and increased isolation.^27^ Perhaps more so than physical illnesses, mental illnesses can erode both the ability and motivation to socialize, making them particularly salient for social isolation research.

Social isolation is a major public health concern. Using the 2022 Mental Health and Access to Care Survey (MHACS) (n = 9861), a weighted, nationally representative sample of the Canadian population, we examine how chronic conditions, chronic pain, and disability were associated with the Social Provisions Scale (SPS-10) scores, a measure of perceived social support.^28^^,^^29^ Social support is a closely related dimension of social relationships that often covaries with social isolation.^5^^,^^30^ The SPS-10 measures social support at both the structural and functional levels, indicating the presence of relationship ties, characteristics of interactions (e.g. frequency), appraisals of support, level of social network integration, and purpose of relationships.^31^^,^^32^ Higher SPS-10 scores indicate greater perceived social support, which is typically associated with lower social isolation.^5^^,^^30^ Thus, while we report results in terms of social support, we discuss implications for social isolation as an associated public health issue. In our analysis, we investigated a disaggregated measure of chronic disease, looking at individual and overlapping chronic conditions in association with social isolation, alongside chronic pain, disability, and sociodemographic controls. We extended population-level research on social isolation with evidence from the Canadian population, and expanded assessments of chronic disease by considering physical and mental conditions simultaneously.

## Methods

### Data source

This study used the MHACS, a weighted, nationally representative, cross-sectional survey collected by Statistics Canada between 17 March and 13 July 2022.^28^ The MHACS had a response rate of 25%, with 9861 individuals out of 39 485 responding.^28^ The MHACS collected data on health status and health service access before/during the COVID-19 pandemic, with numerous variables related to subjective well-being, mental disorder, chronic disease/pain, and disability, alongside a robust list of sociodemographics. Respondents were aged 15 and older from all provinces and territories, excluding: Indigenous peoples on reserve, people in some rural areas, Canadian Armed Forces members, and institutionalized individuals. Data collection was stratified to oversample for Canada’s four largest visible minority groups (Black, South Asian, Chinese, Filipino), and to adequately represent various age (15–24, 25–44, 45–64, 65+) and gender (men, women) demographics. More details on variables and sampling design are available through the MHACS user guide, questionnaire, and data dictionary.^28^

### Variable definitions

Our outcome is the SPS-10, a validated measure ranging from 10 to 40 points of perceived social support that sums respondents’ level of agreement, on a scale of 1 (strongly disagree) to 4 (strongly agree), with 10 questions pertaining to five different factors: attachment, guidance, social-integration, reliable alliance, and reassurance of worth.^29^ The SPS-10 measures social support at both the structural and functional levels, indicating the presence of relationship ties, characteristics of interactions, appraisals of support, level of social network integration, and purpose of relationships.^31^^,^^32^ Higher SPS-10 scores indicate greater perceived social support, which we discuss in relation to social isolation given that it is a common covariate.^5^^,^^30^

We identified three distinct predictors of interest: (1) disability; (2) chronic pain; and (3) chronic conditions. To consider each exposure’s association with social isolation net of other factors, all three were assessed in one analytic model alongside sociodemographic controls.

Disability was operationalized through the 12-item World Health Organization Disability Assessment Schedule 2.0 (WHODAS 2.0), a validated measure ranging from 0 to 40 points that sums respondents’ level of difficulty, on a scale of 1 (none) to 5 (extreme/cannot do), achieving cognitive, mobility, self-care, interpersonal, and daily life activities, where higher scores indicate greater levels of disability.^33^ The WHODAS 2.0 does not reference pain in any questions pertaining to disability, making it distinct from the measure of pain analyzed.

Chronic pain was measured using the Health Utilities Index (HUI) pain-by-interruption-of-activities metric.^34^ Specifically, the HUI Mark 3 (HUI3) classification system was used to construct the variable using two questions: (1) a question asking whether the respondent was “usually free of pain or discomfort”; and (2) a question asking the number of activities that a respondent’s pain or discomfort prevented.^28^^,^^34^ The variable consists of five categories: no pain or discomfort, pain—does not prevent activity, prevents few activities, prevents some activities, and prevents most activities.

Chronic conditions consisted of 10 categories: no identified condition, cardiovascular (high blood pressure, heart disease), asthma, diabetes, arthritis, back problems (excluding fibromyalgia/arthritis), migraine headaches, mental disorders (depression, dysthymia, mania, bipolar, generalized/social anxiety, obsessive-compulsive, panic, posttraumatic stress disorder [PTSD], attention-deficit disorder [ADD]), other unspecified conditions (chronic bronchitis/emphysema/chronic obstructive pulmonary disease, cancer, bowel disorder/Crohn’s/ulcerative colitis, chronic fatigue syndrome, multiple chemical sensitivities, learning disabilities, schizophrenia, psychosis, eating disorders, and other unidentified long-term physical or mental health conditions), and multiple overlapping conditions. This variable was constructed from respondents’ answers to binary (no/yes) questions that asked if they had any of the listed conditions. Categorizations first followed the Canadian Chronic Disease Surveillance System (CCDSS).^35^ Conditions in MHACS that were identical to chronic disease categorizations made by the CCDSS (e.g., arthritis) were left as single-condition categories when they had a large enough sample size to adhere to survey release rules. Conditions that did not match any CCDSS definition were either left as single-condition categories (e.g. migraine headaches) or merged with other conditions when sample size was too small to adhere to the MHACS preweighted sample-size release rules.^28^ Single categories are mutually exclusive. The “other unspecified condition” category merged any conditions with too few respondents to be analyzed alone.^28,^^*^*Schizophrenia, psychosis, and eating disorders were categorized under other, rather than with mental disorders, due to pregrouping by Statistics Canada in the MHACS public use microdata that made recoding these individual conditions impossible. The “multiple overlapping conditions” category includes respondents with more than one condition.

Sociodemographic controls included age, gender, visible minority status, 2SLGBTQI+ status, education, employment status, household income, marital status, immigrant status, region of residence, and religiosity.^28^^,^^34^ Age was recoded to match the target strata of MHACS: 15 to 24, 25 to 44, 45 to 64, and 65 and older. Gender included Men+ and Women+. The plus sign indicates that non-binary respondents have been randomly distributed into each category.^†^†The decision to impute non-binary respondents into each gender category was made by Statistics Canada. The format of the public use microdata file prevented the authors from undoing this change. Visible minority status consisted of not a visible minority (White, Indigenous) and visible minority (Black, South Asian, Chinese, Filipino, other).^‡^‡The decision to categorize Indigenous peoples as not a visible minority was made by Statistics Canada. The format of the public use microdata file prevented the authors from undoing this change. 2SLGBTQI+ status included Non-2SLGBTQI+ person and 2SLGBTQI+ person categories.^§^§This variable categorizes anyone “who is not cisgender and/or whose sexual orientation is not heterosexual under the umbrella term 2SLGBTQI+.” It therefore represents a mix of both sexual orientation and gender identity. This categorization was chosen by Statistics Canada. The format of the public use microdata file prevented the authors from undoing this change. Education was recoded to merge small categories: less than high school, high school or equivalent, college, trades, or apprenticeship, university below bachelor’s degree, and bachelor’s degree or higher categories. Employment status was constructed from a three-category variable that asked respondents their working status.****Statistics Canada, the MHACS public use microdata file, nor the codebooks or user guide associated with the MHACS provide any information on how students were categorized in this data. Worked and absent from work were merged into employed, while “did not have a job” was coded as unemployed. A third category, “excluded,” was added to prevent loss of all respondents above age 75 upon casewise deletion.^††^††Statistics Canada and the creators of the MHACS survey did not ask any employment-related questions to respondents aged 75 and older. When combined with casewise deletion, this leads to the loss of 2504 respondents in the 65 and older age category, cutting the proportion of the sample from about 20% to about 2%. According to the codebooks associated with the MHACS survey, there are 2504 valid skips aged 65 and older in the employment variable (respondents aged 75 and older not asked the question). In the public use microdata file, there are 2507 respondents in the 65 and older age category who are lumped together as missing or NA. This means that three respondents were asked the question but did not answer. In terms of missingness, there are 2504 structurally missing and three non-structurally missing. Due to the way the public use microdata file is constructed, there was no way to identify and separate the three from the 2504. We therefore decided that it is more analytically sound to risk improperly categorizing three respondents, as opposed to removing 2504 from the analysis. As such, the 2507 respondents in the 65 and older category that were missing for employment status were recoded as an “Excluded” category in the employment status variable. Given that employment status is only a control variable with no hypothesized interpretation, we believe this to be an appropriate fix.

Household income in Canadian dollars (CAD) was recoded from 15 categories to: less than $20 000, $20 000 to $39 999, $40 000 to $59 999, $60 000 to $79 999, and $80 000 or more following previous work with MHACS.^36^ Marital status was recoded to merge small categories: married or common law, never married, and widowed, separated, or divorced.^28^^,^^37^ Immigrant status included: non-immigrants and immigrants. Region of residence consisted of rural (< 1000 people), small pop. centre (1000–29 999), medium population centre (30 000–99 999), and large urban population centre (> 100 000). Finally, religiosity measured how important religion or spirituality was to a respondent: not at all important, not very important, somewhat important, and very important.

### Statistical analysis

Following prior MHACS research, missing data for any variables were removed using casewise deletion.^36^^,^^37^ This study has 7994 eligible respondents. Accounting for the complex survey design of the MHACS, survey and bootstrap weights were applied as directed by Statistics Canada.^28^ Weighted bivariable and multivariable linear regression models were run in R version 4.5.2 (R Foundation for Statistical Computing, Vienna, AT), with the latter assessing how all three exposures alongside controls were associated with social isolation simultaneously.

## Results

Table 1 presents the weighted sample characteristics, including the mean and category percentages with corresponding 95% confidence intervals [CI]. The mean average score for the outcome variable social isolation was 35.4 (35.3, 35.6).

**Table 1:** Sample characteristics

**Characteristic**	**Full sample (n = 7994)**
	**Weighted percent (95% CI)**
Age	
15–24	14.4% (14.1%, 14.8%)
25–44	33.9% (33.4%, 34.5%)
45–64	31.7% (31.1%, 32.2%)
65 or older	19.9% (19.5%, 20.4%)
Gender	
Men+	49.4% (48.8%, 50.0%)
Women+	50.6% (50.0%, 51.2%)
Visible minority status	
Not a visible minority	74.1% (73.3%, 74.9%)
Visible minority	25.9% (25.1%, 26.7%)
2SLGBTQI+ status	
Non-2SLGBTQI+ person	93.8% (93.1%, 94.4%)
2SLGBTQI+ person	6.2% (5.6%, 6.9%)
Education	
Less than high school	9.9% (9.1%, 10.7%)
High school or equivalent	24.0% (22.9%, 25.2%)
College, trades, or apprenticeship	29.7% (28.4%, 30.9%)
University below bachelor’s degree	4.5% (4.0%, 5.1%)
Bachelor’s degree or higher	31.9% (30.8%, 33.0%)
Employment status	
Unemployed	20.6% (19.5%, 21.6%)
Employed	61.1% (60.0%, 62.2%)
Excluded	18.3% (17.8%, 18.8%)
Household income	
< CAD 20 000	2.6% (2.1%, 3.0%)
CAD 20 000–39 999	7.3% (6.6%, 8.0%)
CAD 40 000–59 999	11.1% (10.2%, 12.0%)
CAD 60 000–79 999	12.1% (11.2%, 12.9%)
≥ CAD 80 000	67.0% (65.7%, 68.2%)
Marital status	
Married or common law	58.1% (56.9%, 59.4%)
Never married	29.5% (28.5%, 30.6%)
Widowed, separated, or divorced	12.3% (11.4%, 13.2%)
Immigrant status	
Non-immigrant	70.8% (69.7%, 71.9%)
Immigrant	29.2% (28.1%, 30.3%)
Region of residence	
Rural (< 1000 people)	18.6% (17.5%, 19.7%)
Small population centre (1000–29 999 people)	11.2% (10.4%, 12.0%)
Medium population centre (30 000–99 999 people)	8.6% (7.8%, 9.4%)
Large urban population centre (> 100 000 people)	61.6% (60.3%, 62.8%)
Religiosity	
Not at all important	28.1% (26.8%, 29.3%)
Not very important	20.6% (19.6%, 21.7%)
Somewhat important	24.1% (22.9%, 25.2%)
Very important	27.2% (26.1%, 28.3%)
Chronic conditions	
None identified	36.5% (35.2%, 37.8%)
Cardiovascular	5.8% (5.2%, 6.4%)
Asthma	2.6% (2.2%, 3.0%)
Diabetes	1.6% (1.3%, 1.9%)
Arthritis	3.6% (3.1%, 4.1%)
Back problems (Excluding fibromyalgia/arthritis)	4.6% (4.0%, 5.2%)
Migraine headaches	3.6% (3.1%, 4.1%)
Mental disorder	7.1% (6.4%, 7.8%)
Other unspecified	6.2% (5.5%, 6.8%)
Multiple overlapping	28.5% (27.3%, 29.7%)
Pain level	
No pain or discomfort	77.2% (76.1%, 78.3%)
Pain – does not prevent activity	7.1% (6.4%, 7.8%)
Pain – prevents few activities	7.4% (6.7%, 8.1%)
Pain – prevents some activities	5.0% (4.4%, 5.6%)
Pain – prevents most activities	3.3% (2.8%, 3.8%)
WHO Disability Assessment Schedule (0-40) [Mean]	6.3 (6.0, 6.6)
Social Provisions Scale (10–40) [Mean]	35.4 (35.2, 35.5)

**Abbreviations:** 2SLGBTQI+, Two-Spirit, lesbian, gay, bisexual, transgender, queer, intersex, and additional people who identify as part of sexual and gender diverse communities; CI, confidence interval; WHO, World Health Organization.

**Notes:** Percentages may not add up to 100 due to rounding errors.

The plus (+) sign in each gender category indicates that non-binary respondents have been randomly distributed into each category. Decision made by Statistics Canada and the creators of the MHACS.

The 2SLGBTQI+ variable categorizes anyone “who is not cisgender and/or whose sexual orientation is not heterosexual under the umbrella term 2SLGBTQI+.” It therefore represents a mix of both sexual orientation and gender identity. Decision made by Statistics Canada and the creators of the MHACS.

Statistics Canada, the MHACS public use microdata file, nor the codebooks or user guide associated with the MHACS provide any information on how students were categorized in these data.

The analysis of these sociodemographics showed that most participants were age 25 to 44 (33.9% [33.4%, 34.5%]); identified as Women+ (50.6% [50.6%, 51.2%]); not a visible minority (74.1% [73.3%, 74.9%]); not 2SLGBTQI+ (93.8% [93.1%, 94.4%]), had a bachelor’s degree or higher (31.9% [30.8%, 33.0%]), employed (61.1% [60.0%, 62.2%]), with a household income of ≥ CAD 80 000 (67.0% [65.7%, 68.2%]), married or living common law (58.1% [56.9%, 59.4%]), non-immigrants (70.8% [69.7%, 71.9%]), in large urban population centres (61.6% [60.3%, 62.8%]), and perceived religion as not at all important (28.1% [26.8%, 29.3%]).

Our exposures were oriented as follows: mean average disability score of 6.3 (6.0, 6.6), most participants experienced no pain or discomfort (77.2% [76.1%, 78.3%]), and had no identified chronic condition (36.5% [35.2%, 37.8%]).

Results of weighted bivariable (unadjusted) and multivariable (adjusted) linear regression models are in Table 2. Accounting for exposures and sociodemographic controls, only disability was significantly associated with the SPS-10 in the multivariable model (B = −0.09 [95% CI = −0.11, −0.08]). No chronic disease category or pain level was significantly associated with the SPS-10 in the multivariable model.

**Table 2:** Bivariable/multivariable linear regression predicting social isolation (n = 7994)

**Characteristic**	**Bivariable (95% CI)**	**Multivariable (95% CI)**
Chronic conditions		
None identified	Referent	Referent
Cardiovascular	0.11 (−0.35, 0.57)	0.29 (−0.17, 0.75)
Asthma	0.64 (−0.09, 1.38)	0.19 (−0.49, 0.87)
Diabetes	−0.63 (−1.50, 0.25)	−0.32 (−1.17, 0.54)
Arthritis	−0.12 (−0.71, 0.47)	−0.23 (−0.87, 0.42)
Back problems (excluding fibromyalgia/arthritis)	−0.01 (−0.63, 0.61)	0.08 (−0.52, 0.68)
Migraine headaches	0.05 (−0.57, 0.67)	−0.21 (−0.80, 0.38)
Mental disorder	−0.30 (−0.81, 0.21)	−0.21 (−0.72, 0.30)
Other unspecified	0.27 (−0.22, 0.77)	0.17 (−0.29, 0.63)
Multiple overlapping	−0.80 (−1.11, −0.48)***	−0.19 (−0.53, 0.15)
Pain level		
No pain or discomfort	Referent	Referent
Pain – does not prevent activity	−0.19 (−0.65, 0.27)	−0.10 (−0.55, 0.35)
Pain – prevents few activities	−0.76 (−1.24, −0.29)**	−0.33 (−0.81, 0.15)
Pain – prevents some activities	−0.83 (−1.48, −0.17)*	0.12 (−0.50, 0.74)
Pain – prevents most activities	−2.13 (−3.06, −1.20)***	0.22 (−0.72, 1.17)
WHO Disability Assessment Schedule (0–40)	−0.01 (−0.11, −0.08)***	−0.09 (−0.11, −0.08)***

**Abbreviations:** CI, confidence interval; WHO, World Health Organization.

**Note:** Multivariable model controls: age, gender, visible minority status, 2SLGBTQI+ status, education, employment status, household income, marital status, immigrant status, region of residence, and religiosity.

* *p* < 0.05.

** *p* < 0.01.

*** *p* < 0.001.

## Discussion

We assessed the association between chronic conditions, chronic pain, and disability, and the SPS-10 scores. While the SPS-10 is a measure of social support, it covaries with social isolation, which is our focus. The results demonstrated that only disability was significantly associated with the SPS-10 scores in the Canadian population at the time of data collection. While our practical effect size is modest; 12% decrease in SPS-10 score across the range of the WHODAS 2.0, the association is meaningful in a population context, given the high prevalence of functional limitations among Canadian adults.^1^^,^^3^^,^^‡‡^‡‡Practical effect size in terms of percent change is calculated by taking the full-scale change of the coefficient for WHODAS 2.0 and dividing it by the range of the SPS-10. Specifically: [(− 0.09 × 40) ÷ 30] × 100 = 12%. This indicates the difference in the outcome between someone with no disability and someone with the highest score of disability. In order to see the percent change in the SPS-10 caused by a one-point increase in the WHODAS 2.0 scale, the reader should divide the total change (12%) by the range of the WHODAS 2.0 scale (40).These results underscore the importance of focusing on functional capacity rather than diagnostic labels when assessing social vulnerability. Our model highlights the importance of considering different aspects of the illness experience together to understand how the intersection of disease, pain, and disability functions in association with social isolation.

No chronic disease category nor chronic pain level was independently associated with the SPS-10 scores after accounting for other exposures and sociodemographic factors. Rather than indicating an absence of risk, these null findings suggest that the relationship between chronic health and isolation is not driven by diagnosis alone. One possible explanation is that much of the existing research examines conditions in isolation, focusing on clinical or disease-specific outcomes.^38^^,^^39^ Studies that analyze chronic conditions on their own without disability and chronic pain risk obscuring how each condition is associated with isolation. Under these circumstances, omitted variable bias affects coefficient strength, potentially making some conditions appear more strongly tied to the outcome under study. By assessing disaggregated conditions alongside pain, disability, and sociodemographic controls with nationally representative data, we show that the association of many conditions diminishes. Thus, the risk of isolation associated with specific chronic conditions may be less about the diagnosis itself, with disabling consequences and social position being more relevant. Future research should explore these pathways in greater detail.

The absence of an independent association between chronic pain and the SPS-10 scores further suggests that pain may operate on social isolation indirectly through functional limitation rather than serving as a standalone predictor. Pain can restrict mobility, reduce endurance, and limit engagement in social activities, but its social consequences may be most pronounced when it translates into measurable disability. This interpretation aligns with conceptual models that distinguish symptoms from their downstream functional and social effects and highlights the importance of examining mediating pathways in future research. Taken together, these findings reinforce the need to shift theoretical and empirical attention away from disease-specific explanations of social isolation and toward functional mechanisms that cut across diagnostic boundaries. Specifically, future research with more robust data (e.g. longitudinal studies with greater sample size) should explore these mediation pathways.

Disability was significantly associated with social isolation in our multivariable model. This follows trends identified in the literature that emphasize the importance of functional impairment as a driver of social isolation across conditions.^20^^,^^40^ Functional limitations can restrict participation in social activities, create logistical barriers to interaction, and contribute to feelings of burden, all of which heighten isolation. Our results suggest that once disability and other sociodemographic factors are considered, other exposures such as chronic pain may operate indirectly through disability, rather than serving as a standalone predictor of isolation. Policy initiatives aimed at improving mobility, reducing barriers to participation, and expanding assistive technologies are likely to be more effective at reducing isolation than interventions focused solely on managing chronic conditions. From a health system perspective, integrating disability assessments into routine chronic disease care could help identify individuals most at risk of isolation and enable proactive social prescribing or community referrals. Further disaggregation of disability with other measures would yield even more nuanced perspectives as the WHODAS 2.0 does not have a universally agreed upon cutoff indicating disability.^41^

### Strengths and limitations

Our measure of chronic conditions disaggregated conditions individually where possible, while also including both physical and mental conditions, rather than homogenizing dissimilar conditions. This provided a glimpse at (non-significant) associations obscured in previous homogenized analyses. Our results affirm the importance of disability as a central determinant of isolation, which suggests that interventions should prioritize functional supports and accessibility improvements.

Cross-sectional data prevents causal inference; however, the MHACS provides a robust nationally representative picture of the relationship between chronic conditions, chronic pain, disability and social isolation at the time of collection.

More could be done to tease out potential associations between individual chronic disease categories that were not separable in the present study (e.g. cancer).

Casewise deletion and the MHACS pre-weighted sample size release rules limit meaningful subgroup analysis. Future research should hypothesize mediating/moderating pathways in relation to socio-demographics like age, gender, socioeconomic status, and race/ethnicity using more empirically robust data (e.g. longitudinal) and a more direct measure of social isolation.

This survey was conducted late in the COVID-19 pandemic, when patterns of social participation were altered. This may limit generalizability to postpandemic contexts, being informative for understanding how functional constraints shape social isolation under conditions of restricted social engagement.

## Conclusion

This study examined how chronic conditions, chronic pain, and disability were associated with the SPS-10 scores, framed in relation to social isolation among Canadian adults. After adjustment for sociodemographic factors, disability was the only exposure associated with social isolation. This suggests that social isolation is more closely linked to functional limitation than to diagnostic labels or symptoms alone. Analyzing chronic disease, pain, and disability within a single population-level model, this study clarifies that disability and functional limitations restricting social participation/engagement may matter more for social isolation. This distinction has implications for health policy and intervention, underscoring the value of functional assessments in identifying individuals at risk of social isolation. Efforts to reduce social isolation may be most effective when they focus on reducing functional barriers and supporting participation, rather than targeting chronic conditions in isolation.

## Data Availability

None.
